# The Chicago school of ecology’s evolutionary superorganism and the clements-wright connection

**DOI:** 10.1007/s40656-024-00652-4

**Published:** 2025-02-18

**Authors:** Philippe Huneman

**Affiliations:** 1 Institut d’Histoire Et de Philosophie Des Sciences Et Des Techniques, CNRS, Université Paris I Sorbonne, 13 Rue du Four, 75006 Paris, France; 2 Labex Who I Am, Paris, France

**Keywords:** Ecology, Organicism, Group selection, Community, Populations, Synthesis

## Abstract

“Organicism” often refers to the idea that ecosystems or communities are, or are like, organisms. Often implicit in early twentieth century, it has been theorized by Clements, relying on physiological and developmental concepts. I investigate the fate of this idea in major attempts of a theoretical synthesis of ecology in the first part of the twentieth century. I first consider *Bioecology* (1939), by Clements and Shelford, which elaborates clementsian organicism as a general framework for plant and animal ecology. Then I investigate the major animal ecology treatise of the Chicago school ecologists C. Allee, T. Park, O. Park, K. Schmidt and A. Emerson, *Principles of animal ecology* (1949). I show how they shifted organicism from physiology to evolution, synthesizing inspiration from both Clements and Sewall Wright, got their inspiration in evolutionary biology, and built a systematic correspondence between cells, organisms and communities. I claim that the focus on populations allowed them to apply Darwinian insights at the level of communities. Finally I argue that this theoretical synthesis fell apart in the next decade because of the rise of density-dependent accounts of population regulation.

## Introduction

I define “organicism in ecology”, here, as any theory that considers ecological communities or ecosystems as organisms. Many ecologists between 1930 and 1960 were committed to organicism so understood. In his seminal papers that defined an ‘individualistic’ concept of communities opposed to any organicism, Henry Gleason indicated that most ecologists viewed plant associations as analogous to either “organisms” (hence, organicism), or “species”, to the extent that an association is, like a species, made up of variable individuals likely to interact (Gleason, 1939). Against these two views that assume some solidarity and similarity between organisms, Gleason claimed a concept of association according to which “any number of different sequences (of species) may be imagined, all based on variation in the environment and on the species then available through the chances of migration” (Gleason, 1939, p. 100; see Nicolson, 1990). The fact that chance played such a role prevented Gleason from seeing communities in analogy with any kind of structured biological collective, be they species (as evolved interbreeding populations) or organisms (“populations of cells”, as reminded by Emerson in the comments that followed the presentation of the here above-cited paper).

Yet his individualistic concept took over plant ecology much later, mostly in the 1950s. Curtis and Whittaker authored seminal work and especially random sampling of trees and seeds in an ecosystem, which gave plausibility to Gleason’s view (Nicolson, 2001, 2016). Instead of bounded wholes with a history of organic development, plant associations appeared rather as a superposition of migration histories, mostly shaped by chance events and environmental regularities.

In the years between 1926 and 1951, namely between Gleason’s critique of community concepts of associations and the adoption of the individualistic concept by a major share of ecologists, in plant ecology and more generally ecological communities were easily understood in organismal terms—a matter of fact denounced in Gleason’s, 1939 retrospective paper. One can plausibly argue that this period witnessed the elaboration of sophisticated organicist concepts of communities and ecosystems—these concepts themselves being in the process of naming and acceptation. The present paper will address the evolution of ecological organicism in this period.

In order to grasp the organicist idea’s main lines of unfolding, I will concentrate on two groundworks that had the ambition of synthesizing the ecological knowledge of their time, namely Clements and Shelford’s *Bioecology*, and the well known treatise *Principles of animal ecology* by Warder C. Allee, Thomas Park, Orlando Park, Karl Schmidt and Alfred Emerson, published in 1949 (hereafter PAE). All authors listed here had prominent institutional and educational roles in the community of ecologists, and their endorsement of organicism in the reference works just mentioned is significant for our understanding of the history of ecology. Even though these books were ‘textbooks’, they didn’t sacrifice depth and exhaustiveness in favor of pedagogical accessibility. Concerning the PAE, Charles Elton—the author of the landmark book *The ecology of animals* (Elton, 1927)—said in his review that “It is by far the most important treatise on the subject that has ever been published,” a praise that does not characterize a textbook intended only for teaching.

I will center on the *Principles*, and I will question the continuity between it and *Bioecology* regarding the meaning of organicism. I will argue that the view of communities presented in the *Principles* synthesizes Sewall Wright’s views on natural selection and Frederic Clements’ ideas on the development of communities, and that is shifts the latter away from a physiology-inspired view to an evolution-oriented understanding. In a word, I will argue that with this groundbreaking treatise community ecology shifted from a physiological to an evolutionary organicism. Even though some of my arguments will focus on the introduction of evolution in the tradition of organicism in ecology, which will draw upon some of the views exposed in Huneman (2019) and reinforce some of its conclusion, the overall ambition of this paper is to contribute to a history of organicism, rather than of evolutionary thinking in ecology. I will also examine how this Clements-Wright connection faded away though the demise of group selection as advocated by the *Principles*, and what were the consequences for theoretical ecology.

I will start by presenting the divide between individualism and holism at the times of Elton’s *Animal ecology* (1927), and by sketching the most general ideas constitutive of organicism. Then I will turn to the major synthesis published in the beginning of the next decade, Clements and Shelford’s *Bioecology*, in 1939, and analyze their organicism. The third section will consider in detail the ecological “organicism ” of the *Principles*, trace some of its sources, and argue for the above mentioned notion of a “Clements-Wright connection ” present in this work—as well as expose its fading away.

## Individualism and organicism in early 1920-30 s ecology

Ecology constituted itself as a scientific discipline in late nineteenth century by taking its inspiration from evolution, after a long time where evolution had been a model to follow, for instance when Eugene Warming (1909) was doing plant ecology (Coleman, 1986). Later on, the major reference for ecologists became the experimental methods of physiologists, with Frederic Clements, or ecologists such as Thomas Park and Georgii F. Gause. According to Ilerbaig, (1999), Victor Shelford, who became the first president of the Ecological Society of America in 1915, promoted physico-chemical reductionism and experimental manipulation as a way to strengthen the scientific character of ecology. All turned to more robustly scientific perspectives; when it was not physiology, it was mathematical modeling, as were doing Gause, Lotka, Volterra, Nicholson (Kingsland, 1995). Ecology in early 1930s featured at least four major cleavages, that run through its subsequent history. Some ecologists focused on mathematical models such as Lotka and Volterra equations, or the models of population regulation by Nicholson, (1933) and Nicholson and Bailey (1935). Others were more inclined towards observations and experiments, for instance Thomas Park’s experiments on *Tribolium* (Park, 1948) or Allee’s on goldfish, which investigate the effects of population size increase (Allee, 1931; Park, 1948). Hagen (1989) importantly notes the existence of two traditions in ecology from the 1920s on—that are irreducible to each other but belong together to the nature of ecology, a claim I will however not address here—namely “holological ” and “mereological” ecology (as Hutchinson called them before Hagen). The former considers communities or ecosystems as wholes, with a kind of cohesion and general behavior that is sui generis and should be approached as such. The latter considers those systems from the viewpoint of their parts, for instance individual organisms and species; it is exemplified by the individualistic concept of community (Gleason 1926, 1939) but consists in a much more general approach to ecology. In addition, Hagen reminds us that both approaches could be more or less oriented towards evolution.^1^

*Hololo*gical approaches include varieties of ecological organicism. Most generally, ‘organicism’ is part of a set of pairs of concepts that are intended to specify relations between understanding of elements and understanding of systems, and, at the same time, characterize the ontology of the relations between systems and their internal elements. At least, among this class, one finds the following oppositions: reduction/emergence, individualism / holism; mechanism / organicism (or mechanism vs. finalism).^2^

A short characterization of these distinctions allow us to philosophically situate ecological organicism. Reduction means that the understanding of a system somehow derives from the understanding of things at a lower level, e.g. its elements and, additionally, that the high level behavior of the system results integrally from the sum of the low level behaviors. Emergentism in turn rejects either this derivation or this resulting (e.g. Wimsatt, 2006; Sartenaer, 2016; Huneman, 2008; Humphreys, 2016). Holism/individualism is about the relation between individuals that stand at the low level, and the system, which is the whole of all individuals: individualism stipulates that the key for understanding the whole is the individuals and their behaviors—while holism ascribes to the whole itself the explanatory power to make sense of its elements (see Epstein, 2018). Mechanism is rather about the functioning of the parts and of the whole—Kant famously saw in ‘mechanism’ the claim that the explanation of the whole comes from the explanation of the parts, while Cartesians used to see mechanism as the commitment to the claim that everything is governed by the laws of Cartesian mechanics (Kant, 1791; Huneman, 2007b). Finalists claim in contrast that the behavior of the parts has to be understood from the whole—some finalists being (like Kant) neutral regarding whether finalism is ontologically founded.

Then, in the space between finalism and holism, ‘organicism’ comes into play. An organicist biologist indeed means rather a holist with respect to living beings; the kinds of connections postulated between the “purpose” proper to finalism, and the “whole ” proper to holism, defines organicism and its varieties. But generally, organicism is more specific than holism, since it interprets the relation between parts and wholes as relations between organs and a whole organism, therefore in a *functionalist* way: organs have *functions*, which can be seen as their contributions to the survival and reproduction of the whole.^3^ For this reason, ‘organicism’ is a kind of holism very close to finalism.

Seeing ecological communities as something more than sums of species interacting and as more than individuals locally interbreeding, as Clements or Elton did, minimally entails a commitment to some emergentism and to some holism. It needs not imply any finalism or organicism: not all systems and wholes are organisms (see also Nicholson and Gawne (2015) on organicism). Therefore the claim that communities are organisms or like organisms should be set apart from other kinds of ecological holism.

In a retrospective paper defending his 1926 formulation of the individualistic concept of plant association Gleason wrote: “The individualistic concept is totally at variance with the idea that the association *is an organism,* represented by many individuals, and also does not admit an analogy or homology between *the species* and the association. While affirming the existence of definite communities, characterized by reasonable uniformity over a considerable area terminated by a definite boundary, the concept *denies that all vegetation is thus segregated into communities.*” (Gleason, 1939, my emphasis).

Interestingly, Gleason (1939) acknowledges here that there are several anti-individualistic, hence holistic, concepts of communities. Only one is “organicist,”—and typically reminds us Clements’s ideas about communities—while the other one is species-like and refers to the phytosociologies of Braun-Blanquet.^4^ Indeed textbooks oppose him to Frederic Clements, who advocated a view of communities as having a development of their own, following laws that apply to the wholes and which have to be unraveled by ecologists. Eliot (2005) provides a more nuanced view of Clements, debunking the kind of ‘romanticism’ often associated to his views, even though the textbook contrast was rhetorically useful.^5^

Kirchhoff (2020) provides useful further characterization of Clements’ organicism by distinguishing two modes of organicism: the mutualistic view according to which an organism is a set of many organs interacting functionally; and a hierarchical view, in which the key relation allowing one to make sense of the link between parts and wholes is the hierarchical relation. (This distinction is orthogonal to the kinds of holism distinguished by Gleason (1939).) Applied to ecology, the latter means that some species are dominant while others are subordinated. This emphasis on the dominance relation sets Clements apart from a very old tradition of highlighting the wholeness of ecosystems (Reill, 2005), in sense of the reciprocal usefulness of all parts of it. It accounts for the fact that Clements would, as other more individualism-leaning ecologists, in practice experimentally look for pairwise subordination relations, in line with Shelford’s call for experimentation and causal reasoning.

Clements was essentially a plant ecologist, working with the key concept in this domain, namely the plant association or formation. However, the key role of dominance relations might be found in animal ecology at the same time. In *Ecology of Animals* (1927),^6^ Charles Elton indeed addressed the issue of the regulation of species abundances in multispecies communities, and the reasons explaining the composition of communities, such as trophic chains; these are clearly dependence / domination relations.

Dussault (2020) makes clear how Elton is not a full-blown organicist, in any sense. He pays attention to the repeated metaphor Elton uses, namely the relation between an ecological community and a society. Organisms are much more integrated than societies (at least the ones we spontaneously think about, the large vertebrates), therefore the ‘social community’ is a kind of holism devoid of organicism. Epistemologically, Elton makes along these lines the important claim that animal communities are “subject to definite *economic* laws” (viii, my emphasis). Thus, for him the laws that govern ecological communities are not physical laws, neither are they biological laws, but they are *economical*—since they mostly concern division of labor, competition, supply and demand of resources and circulation of matter, and their unraveling requires experimental and quantitative methodologies.

Hence at the beginning of the 1920s we witness at least three brands of holism: a mutualistic organicism, inherited from German romantic biology (see Esposito, 2014; Richards, 2002); the dominance-organicism, represented by Clements (1916), and the weak, economically minded, organicism which Dussault attributes to Elton.

But how will those Eltonian “economic ” laws be determined? This raises the crucial question of what communities should be, if those laws are to hold. More importantly, should they really be economic or social, as Elton states, or rather inspired by organismic functioning? One can see the major attempts to provide a general theoretical framework for ecology, in the three forthcoming decades, as answers to this question regarding the proper nature of holism adapted to ecological science. In what follows, I will investigate how two kinds of organicism have been proposed as answers to this question, and in which sense they amounted to such answers.

I will first focus on Clements and Shelford’s treatise *Bioecology* (1939), and then turn to Allee and colleagues’ key book published ten years after, *Principles of animal ecology.* Both books attempt to achieve a synthesis of ecological knowledge framed into a general theory of ecology. Both seem to offer a type of organicism. I will explore those two types and study the shift from one to the other in the next two sections. The last section will comment on the subsequent demise of such organicism.

## Clements and Shelford’s bioecology: organicism as a development- and coaction-based framework

*Bioecology* (1939; hereafter CS) by Frederic Clements and Victor Shelford, is an attempt to provide a unified theoretical framework for plant and animal ecology. Both authors were renowned ecologists at the time of its publication. Clements, ecologist at the Carnegie institution in Washington DC, was already very influential for his approach to plant ecology, using novel concepts and a set of neologisms—famously, the biome, the sere and the climax – which served to describe the stages of a developing community, namely the ecological “succession ” (Eliot 2005; van der Valk, 2014). Shelford was a prominent animal ecologist, who started with terrestrial animals and then turned to marine ecology. He graduated at the University of Chicago in 1907 and then spent his career nearby, at the University of Illinois, after 1911. He authored an important book, *Animal Communities in Temperate America* in 1913, where he tackled ecological successions in some animal communities (Benson, 1992). Clements and Shelford’s common book was intended to generalize key concepts of Clementsian ecology to marine and aquatic environments: “A signal extension of ecological ideas is involved in the application of climax and succession, that is of development, to lake and ocean.” (CS, 4) More generally, *Bioecology* intended to unify ecology, especially by integrating plant and animal ecology. This does not negate what plant ecology is, namely an investigation of the relations between plants and their environment, but it mainly complexifies it.^7^ It implies a shared uniformity of methodological practices, beyond the ontological distinctions.^8^

The book credits Forbes’s 1887 essay on “The lake as a microcosm” for having first expressed the idea of ecological systems as organisms. But the crucial concept of *biome* or biotic community has been accessed independently, as Clements and Shelford say in a long initial historiographic chapter: the “distinction of the biome as the basic concept in climax and succession” came in 1916 through Clements’ view, according to which “plants were considered to exert the dominant influence, although it was recognized that this role might sometimes be taken by animals. The biotic community is fundamentally controlled by the habitat and exhibits both *development* and *structure*. In its development the biome reacts upon the habitat and thus produces a succession”(CS, 6–7).

I highlighted in the last quote the key ideas of *development* and *structure*: having these internal properties turns communities as sets of species into something like organisms. Clementsian novel concepts such as the “sere ” are understood throughout this analogy with development: “The sere represents the cycle of development of the community, which resembles in many respects the life cycle of the species-individual.” (CS, 27). Thus, *Bioecology* emphasizes this key role of development in ecology: “Development is the basic process of ecology, as applicable to the habitat and community as to the individual and species (Clements, 1904, 1905). It recognizes that life constitutes a dynamic system and that static studies are valuable only as they throw light on development or serve some practical purpose in this connection.” (CS, 3) Clements’ other newly forged notion, the biome, instantiates here organicism, due to acknowledging the roles of succession and development: “The concept of the biome is a logical outcome of the treatment of the plant community as a complex organism, or superorganism, with characteristic development and structure. As such a social organism, it was considered to possess characteristics, powers, and potentialities not belonging to any of its constituents or parts” (CS, 20). The sociality rather indicates here Elton’s weak organicism, defined by social relations of domination between elements.

The connection between plant and animal ecology is presented in CS as achieved by Shelford around this generalized notion of development: he “elaborated the developmental relation between plant and animal communities (1911–1912)”. The articulation between ecology and embryology then proceeds from the concept of ‘succession’, previously introduced by Clements.^9^ Thus, *Bioecology* cites a set of researches that developed the concept of biome in exploring various cases of successions. Especially, Shelford’s studies have applied with some success the clementsian ideas of biome to marine animal communities.^10^

According to Allee et al. (1949), Shelford’s work on succession, published in numerous articles, was indeed groundbreaking, even though in “present day ecology succession no longer occupies so prominent a place. It is studied but the emphasis is the total community, with succession essentially a developmental phase of the total unit.” (PAE, 54) Interestingly, they noticed that Shelford went “away from evolution as an interpretative factor in ecology and towards physiology and function”. (ibid, see Mitman, 1992, pp. 39–40; Ilerbaig, 1999, pp. 456–57)^11^ This concurs with the eclipse of evolution as a reference point for ecologists, which was mentioned through Coleman (1986) above.^12^ Even more importantly, Shelford’s historical narratives about ecological succession provided a rationale for the enterprise of *Bioecology*, to the extent that the book defends a kind of organicism based on Clements view of communities, according to which ecological successions could be understood as biological development.

To specify this organicism, they write:

“One of the first consequences of regarding succession as the key to vegetation was the realization that the community, as noted above, is more than the sum of its individual parts, that it is indeed an organism of a new order (Clements, 1901, 1905). For this reason, it was *considered to be a complex organism, bearing something of the same relation to the individual plant or animal that each of these does to the one-celled protophyte or protozoan.* The novelty of this proposal naturally evoked criticism, but in spite of this the concept has slowly grown in favor, with dynamic ecologists in particular, and by an increasing number has come to be regarded as constituting a new basis for almost unlimited development (Jennings, 1918). However, *it is essential to bear in mind the significance of the word "complex" in this connection, since this expressly takes the community out of the category of organisms as represented by individual plants and animals.*” (CS, 21, my emphasis).

Thus, strictly speaking, communities are not organisms, in the same way organisms like plants are not unicellulars, even though their functional organisation bears major analogies with the one of unicellulars or, as they say, protozoans. The CS organicism is an analogy *stricto *sensu^13^: the community bears with the individual organisms the same relations these organisms have to protozoans. The reference to ‘complexity’ acquires a double logical meaning: it captures the relation that is common to those two pairs of concepts; and it sets ‘communities’ aside from organisms since they are more complex. Clements and Shelford situate their concept in the context of numerous authors who entertained similar views: Jennings, importantly Phillips, and many of the students of social insects such as Wheeler. They also philosophically characterize their view as ‘holism’, referring to the recently published book of Smuts. Moreover, they see ‘emergent evolution’ sensu Broad or Morgan (whom they cite) as the “most significant contribution to the concept” (p. 23). Yet the ‘evolution’ in ‘emergent evolution’ has nothing to do with Darwinian evolution since it does not appeal to natural selection.^14^

Thus, in the family of concepts indicated before that epistemologically shape the parts/whole distinction, they subscribe to both holism and emergentism, and characterize the whole as an integrated system through coactions. They found in Smuts’ book an acknowledgement of ecology as the key locus of holism, quoting Smuts’s following sentence: “the new science of Ecology is simply a recognition of the fact that all organisms feel the force and moulding effect of their environment as a whole”. Biomes are a main instance of entities that require holism. The molding effect of environment corresponds to the weight placed by CS on the ‘habitat’, as exemplified in this statement: “the essence of ecology lies in its giving the fullest possible value to the habitat as cause and the community as effect, the two constituting the basic phases of a unit process”.^15^

They insist, in fact, on the complexity of causal relations constituting ecology in which it’s not enough to study only one term, the habitat. “Inherent in the very name itself is the basic principle of ecology that the habitat is the complex of factors or causes. Ecology is not merely the science of the habitat, but peculiarly also of the cause-and-effect relation between this and the biotic community, whether on land or in the sea” (p. 23). This leads one to redefine the concept of habitat, by narrowing it down to abiotic elements which become ‘factors’, while plants and animals respond to these factors through complex causal relations: “With plants and regarded as essential constituents of the community, it becomes undesirable if not actually misleading, to refer to either as biotic factors. The word factor should, in consequence, be restricted to the various physical forces or conditions that constitute the habitat” (p. 26). One should note here that this anticipates the position of the champions of density-independent, hence abiotic, factors in the debate on the regulation of species.^16^

But how is this ‘complexity’ of the community to be explained? One can start with a key notion to make sense of organisms in a biome, namely “coaction”. *Bioecology* devotes a chapter to “coaction ” defined as such: “in its simplest form, each coaction comprises the reciprocal behavior of two individuals of the same or different species; the more complex coactions involve the interaction of one group or community with another”. (CS, 104) Animals and plants are coactors together, something that the division of plant and animal ecology previously made impossible to understand (CS 21). Coactions range on all aspects of life but “the most universal of coactions are concerned with shelter and food, directly or indirectly”(ibid).

The notion of coaction allows *Bioecology* to distinguish two opposite outcomes that will inversely affect the community and receive two distinct names, “cooperation” and “disoperation”, defined by the net effect of coaction, since “the same process frequently produces both helpful and harmful effects”.^17^ “When the first outweighs the second, the result is *cooperation* in some measure; if the scales are reversed, the outcome may well be termed *disoperation*.” They are therefore two contrasting outcomes of coaction, which entail opposite consequences for the community. Through coaction, an “organic ” integration of animal and plants is possible, hence the crucial importance of coaction for the community as organism.^18^ Coactions are integrative, and their aggregation account for the holistic response to habitat that defines a community; one may wonder why Clements and Shelford would then jump to organicism, since holism could be enough; but the dimension of development, which is so central in Clements’s succession studies, allows for this step.^19^

The coaction concept is more general and abstract than mere predation or feeding. In their retrospective chapter, Allee et al. (1949) noticed that for ecologists in the early twentieth century, “communities are integrated to a large degree by the sum total of their feeding relations and these relations are the common property of all communities” (PAE, 59). Thus, speaking of coaction, Shelford and Clements, among some others, allowed one to jump to a concept much more general than ‘feeding’ in order to understand communities’ maintenance and structuring. Accordingly, crowding will be, for Allee and colleagues, a form of coaction (see below) as well as “environmental conditioning”, namely the “modification of the effective environment by the population-group activity” (PAE, 352^20^).

But already in *Bioecology*, “aggregation” is a major vector of coaction.^21^ In this viewpoint, the outcomes of aggregation-based coaction are threefold. “Two of these, cooperation and competition, are well known by name, but still too little understood as to fact; the third deals with processes indirectly harmful and may consequently be termed disoperation” (CS, 149).

Cooperation is described among animals and plants. *Bioecology* refers to Wheeler’s “Social life of insects” (1911), and to Allee’s studies on aggregation and crowding—of which I talk below—as well as to Kropotkin’s famous essay on “mutual aid”. However, the essence of competition “is the attempt to secure more than a proportionate share of a limited supply of something, e.g., raw materials, food, space, or material for construction. In comparison, disoperation includes chiefly those harmful effects that have to do with changed conditions or behavior, as in the accumulation of carbon dioxide, toxins, or excreta” (CS, 157). Competition is to be distinguished from all other co-actions by the test of a common demand upon a limited supply (CS, 160).

Thus, with respect to individuals, destruction is the typical outcome of food coactions, though under some conditions coaction will hold ecosystems together. The question, for ecologists, is: when is disoperation swamped by cooperation? Only this outcome allows communities to be maintained and thrive. Dominance relations between species therefore emerge from the results of coaction; they might vary, with dominated species being deflated by novel ones, while the community persists because the disoperation remains overall swamped.

Then, through this notion of coaction *Bioecology* addresses the coexistence question—“which species can coexist with which locally?”. Yet it doesn’t introduce any quantitative or formal treatment. The general view proposed by this treatise contrasts here with Nicholson’s papers on population regulation, which elaborated a mathematical model to study regulation (Nicholson, 1933; Nicholson & Bailey, 1935). In an incisive review, Hutchinson (1940) criticized such lack of mathematical modeling; he saw the whole CS enterprise as descriptive and classificatory rather than explanatory; if a community is an organism, as is stated in the book, the ecologist should provide means to explain the metabolism of such entity, which is not done.^22^

Ten years later, in the *Principles of animal ecology* (1949) the language of coaction and disoperation would remain, as well as the question of their confrontation, even though authors would try to venture into a more explanatory theory. But the solution gives up the developmentalist schemes used by Clements and Shelford; explicit analogies between organisms and ecosystems are given but they are now rooted in natural selection and evolution.

## Looking for new principles: the Clements-Wright connection in the Chicago school of ecology

The *Principles of animal ecology* was immediately recognized as a landmark in ecology. Dobzhansky reviewing it acknowledged an unrivaled attempt to set evolution within ecology: “If the book under review were to accomplish nothing more than to persuade ecologists that the above propositions are true, its important role in the history of ecology would be assured”. (Dobzhansky, 1950) Elton, already quoted, notices that “it is by far the most important general treatise that has ever been published on the subject” (Elton, 1950, p. 74). The continuity with Clements and Shelford’s work is acknowledged in the *Principles*, which cites it over fifty three times.

First, a few words of context. Its authors were confirmed biologists. All of them were from Chicago—Clyde Allee, Alfred Emerson and Thomas Park from the University of Chicago, Karl Schmidt from the Field Museum and Orlando Park from Northwestern. Emerson, Thomas Park and Allee presided the Ecological Society of America, and Emerson and Thomas Park were editors of the journal *Ecology* (1932–1939 and 1939–1949 respectively)*.*

Clyde Allee was a student of Shelford, who supervised his graduate work in 1911. He was hired at the University of Chicago in 1921, and still worked with Shelford, publishing with him and Orlando Park the *Laboratory introduction to animal ecology and taxonomy* (1939). He extensively wrote on social animals and crowding, and forged the concept now labeled “Allee effect ” (see below). A major academic actor for decades, he was an editor of the prominent journal *Ecological monographs* as well as editor of *Physiological zoology* for twenty-five years (Caron, 1977).

Alfred Emerson was a termite expert, hired at the University of Chicago in 1925; he was interested in evolution, and has been a president of the Society for the Study of Speciation, created in 1936, which foreshadowed the society for Common Problems of Genetics and Paleontology, in the context of which the journal *Evolution* has been launched (Smocovitis, 1994; for Emerson’s role, see Cain, 1994).

All five authors from the group had a history of working together: Schmidt and Allee published the book *Ecological Animal geography* 23 with Hesse (Allee et al., 1932); Thomas Park started under the direction of Allee an important series of experiments on flour beetles (Park, 1948). Emerson was a friend of Schmidt, who was also friend with Allee; once at Chicago, where Emerson arrived in 1925, he became acquainted with Allee (see Mitman, 1988 on his relation with Allee).

William Morton Wheeler was an expert on the behaviour of insect colonies, and author of the seminal paper on colonies as “superorganisms” (Wheeler, 1911). Emerson spent some time with him when he did field work in the Tropical station of Kartabo in British Guinea in 1910, and was enthusiastic about this encounter—later on Allee met him at Chicago while working with Shelford (Schmidt, 1957)—and did his necrological notice in 1937. Wheeler’s essay on superorganism—which coined the term and used it for colonies—is often referred to by Allee and Emerson, and then in the *PAE*. Theoretically, Wheeler was close to those emergentist philosophers that CS view as the closest to their organicism, namely Alexander and Broad.^24^ The conception of selection targeting groups, that is prominent in PAE as we will see, has been durably influenced by the connection with philosophical emergentism, which was shared by many influent evolutionists such as J. Huxley (Gibson et al. 2013, p. 511).

In his retrospective memoir about Emerson, Schmidt recalls about Allee and his four colleagues: “They recognized that the time was ripe for formulating principles of ecology, and started to meet in an Ecology group weekly at Chicago” (Schmidt, 1957). In 1938 Allee and Park wrote a reflective paper on ecology: “Concerning ecological principles” (later published in *Science*), that they read to the group, and which triggered the project of a book on the principles of ecology. The ecology group discussed all aspects of theoretical ecology in Allee’s house, and was in 1942 invited by Saunders publishers to write a reference book, which would become PAE.

A specificity of this work was the inclusion of an explicit evolutionary perspective. Actually, Dobzhansky, and also, as we shall shortly see, Ernst Mayr and Sewall Wright read the first drafts of many sections of the book, and Wright and Dobzhansky’s views are often extensively recollected there, for instance about adaptation (Huneman, 2019). Being Chicago-centered, while Wright made almost all his career as evolutionary biologist at the University of Chicago, the group at the times often underwent the influence of Sewall Wright’s views on evolution. Wright had been a key actor since long in the department, he constantly discussed with these ecologists and intervened sometimes on mathematical aspects of their models, especially statistics. Besides, Emerson’s involvement in the societies that would become the Society for the Study of Evolution, and his ties to Ernst Mayr (Cain, 1994), attest that the group responsible for this treatise was constantly interacting with the building of evolutionary biology at the same time.

The book is structured into four sections, which follow a long historical overview, on “environment’, “population”, “community”, and finally “ecology and evolution”. While it introduced evolution, it shares the same ambitions of a synthesis of ecological knowledge that drove *Bioecology* ten years before. It keeps from Clements the idea that ecology should deal with supraorganismal levels of cohesion. Yet, emphasizing the difference between ecological issues and current physiology (referred to in CS), the authors write: “*for ecology*, the supra-individualistic units are *real entities*. Aggregations, populations, societies, and various units at or near the community level present problems rarely recognized by physiologists working as physiologists” (PAE 2, my emphasis).

The book takes up Clements and Shelford’s question on coaction and whether disoperation can be trumped by cooperative processes in communities, so that the integration and cohesion of the community is ensured, and hence the coexistence of species. “Coactions” is still a structuring concept of the section on populations, and its three possible outcomes, cooperation, disoperation and competition, combine in determining the dynamics of populations and communities.^25^

This worry about disoperation vs. coaction—and the notion of ‘disoperation’ itself—would remain active in ecology for long. “Disoperation” is still a major concept of animal ecology several decades after; in 1961, Kendeigh in his *Animal ecology* cites the *Principles* when he writes: “Between organisms, coactions that are beneficial to one or more of the participants constitute *cooperation* (Allee et al., 1949). Cooperation may occur between members of the same species or of different species. (…) As opposed to cooperation, coactions that are harmful to one or more of the participants constitute *disoperation*.” (Kendeigh, 1961, p. 174).

For all these reasons it seems plausible to talk about ecological organicism in the PAE. I will first explain what are the conceptual inputs from the four authors who built this organicism, whose roots are Clements’ idea of communities and Wright’s evolutionary theories, as I will establish. Then, in the light of those explicitations I will interpret the connections between organisms, populations and cells, exposed by the *Principles* in a Table (PAE, 440). I will be relying on Huneman’s (2019) analysis of the evolutionary synthesis proposed in PAE and ecology more broadly, but my focus here is organicism, rather than evolution. While Huneman (2019) attempted to show how the PAE plays a role in the history of acclimation of evolution into ecology, my object here is their role within a history of organicism in ecology. Evolutionary concepts are important there because they were essential to the authors’ approach to organicism, but my analysis is rather a contribution to our understanding of the relation between holism and mereology in ecology, and, more precisely here, of how the lexicon of coactions elaborated in CS shifts towards another explanatory scheme for organicism.

### Prior seminal contributions: Allee, Emerson and Thomas Park

#### Allee and Park

Clyde Allee’s previous work in the 1920s heavily focused on groups of organisms; he studied the “social life of animals” extensively. From various angles he made the point that the grouping of organisms is not always deleterious, or, more precisely, that crowding can have a positive effect until it exceeds some amount of overcrowding. Such positive returns from crowding have been then called ‘Allee effect’.

As Kendeigh summarizes, Allee shows that the aggregation of organisms has changing effects that shift from cooperation to disoperation after a particular threshold is reached. “The beneficial effects of aggregation are lost if the aggregation is either too small or too large (…) Smaller densities are unable to control the growth of the yeasts on which [drosophila] feed; greater densities exhaust the food supply and excessive amounts of excreta accumulate.” (Kendeigh, 1961, p. 175).

**Fig. 1:**
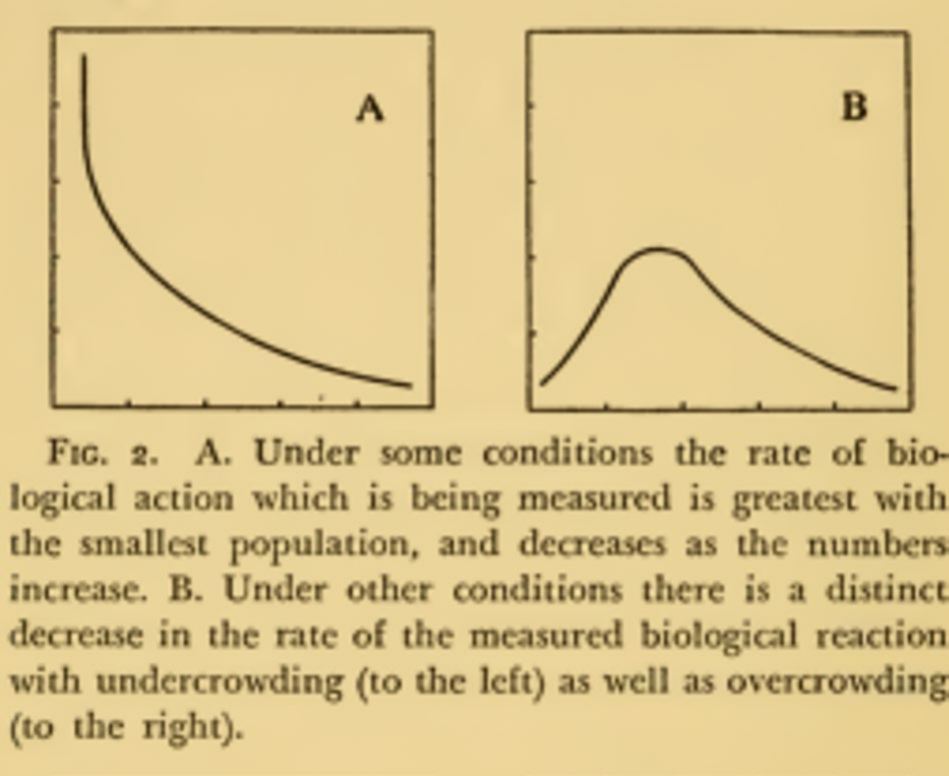
Effect or crowding according to Allee (1938) (Note that units of vertical axis are undefined)

Identifying this “Allee effect” is a contribution to a major issue for ecologists, namely the regulation of population numbers in a species. Allee mostly conducts experiments, and then, borrows others’ concepts to make sense of these experiments. He initially viewed his study of aggregation as “contribution toward the development of general sociology upon a physiological basis”, sharing here his teacher Shelford’s commitment to physiology (1931, p. 3). His three books—*Animal aggregation*, 1931, *Animal life and social growth*, 1932, *The social life of animals*, 1938—synthesizing his work on crowding include the results of these experiments. They previously appeared in a series of papers published from 1926 to 1931, under the general title “Animal aggregation”—where he gave reviews of existing observational and experimental knowledge. He also provides verbal theorizing which only rarely includes mathematical formulations, apart from some diagrams showing the curve of the Allee effect such as Fig. 1, which does not include units of measure. *Crowding* is for him a major feature of animal life because, as evidence seems to indicate, “animals are rarely solitary; (…) they are almost necessarily members of loosely integrated racial and interracial communities” (Allee, 1938, p. 38).

Crowding is indeed therefore the “simplest start toward social life which is easily apparent and a condition of nearly all society,” (ib, p. 31). Hence its study contributes to the understanding of group dynamics and animal behavior.^26^ This allows the establishment of a “general principle of automatic co-operation which is one of the fundamental biological principles”. Allee’s interest is in showing that, while crowding may have detrimental effects, in many conditions (conditions “B” in Fig. 1), some crowding has as effect a non linear increase in survival value—until this effect is swamped by overcrowding. Simply put, his question is then: “given the evidence at hand, that optimal numbers present in a given situation have certain positive survival values and some definitely stimulating effects on the growth of individuals and the increase of populations, we strike the problem of the optimal size of a population in nature.” (ib., p. 49).

One experiment, an early work by Allee, compares goldfish in water where others have lived, and goldfish in clean water: the former grow better (1938, p. 94). Another example was Thomas Park’s (1934) experiments on flour beetles, supervised by Allee, which showed purposely that increased density increases to some extent the reproductive output of these organisms, possibly by stimulating copulation.^27^

Crowding is a “proto-cooperation”, and Allee saw cooperation as an instance of a basic principle, “a fundamental trait of living matter”, expressed in animals without even the requirement of consciousness (which, for Allee, was at the times ‘discarded’ by psychologists) (Allee, 1931, p. 355). This cooperation principle combines with a more Darwinian principle to govern the biological domain: “two ideas explain the order that is evolved in such communities. First, there is the background of common interests and of unconscious co-operation among all the elements of the community, the nature of which will be discussed later. Second there is the struggle for existence and the elimination usually of the less fortunate but, at times, of the less fit animals” (Allee, 1932, p. 39). Importantly, here Allee sets Darwinian evolution by selection in opposition to this other principle, that he sometimes saw as the expression of an instinct towards sociality—an “unconscious cooperation”—here, realized at each biological level of complexity.^28^

But interestingly, in his 1938 book, Allee counted Sewall Wright’s views among the possible mechanisms for his Allee effect.^29^ First, he understood Wright’s notion of drift (Wright, 1931) in a way that supports his idea of the beneficial effects of crowding: according to the meaning of drift, Allee considers that increased population sizes come with an increase in the intensity of selection, and therefore, that crowding yields adaptation on an evolutionary scale. In small populations “fixation will be a matter of chance, and local races will result w*ithout any necessary reference to adaptation*” (Allee, 1938, p. 123, my emphasis).

Second, he also appeals to Wright’s major theoretical view, namely the “shifting balance theory” (SBT).^30^ Chapter IV—which has been checked by Wright himself—includes diagrams from Wright, including some of the SBT. For Allee, the upshot is that even though the increase of population size is good for adaptation, the optimal size of population is not the largest one, because cleaving the population into groups of mild size allows this SBT process to enact a more efficient adaptive evolution. What interests Allee is that, like in the case of crowding, the intermediary population size is the best one—because small populations are such that adaptation isn’t reached and extinction is faced, while large populations may be stacked on a non-optimal adaptation. This is exactly what Wright saw when he highlighted the problem of populations stuck on local fitness peaks, which led him to the SBT as a solution: if there are several fitness peaks, each representing several local fitness optima in a fitness landscape, and a population is on a hill that has a low fitness peak, natural selection alone will drive it towards this local peak and the population will remain stuck there, unable of reaching the global fitness peak, which represents the genuine adaptation in its environment.^31^

Wright’s SBT matches the lessons of Allee’s lab and field experiments about crowding and population size, namely that “the optimal population size does not coincide with either the largest or smallest possible but lies at some intermediate point” (id., p. 132).^32^ Allee’s work was indeed widely read and criticized by his fellow ecologists at Chicago, especially, and by Sewall Wright himself.^33^

Notice that Allee’s constant focus is ‘aggregation’, namely the pure effect of numbers; it directly connects with the population-level processes that are evolution and selection, as the reference to Wright makes it clear. Yet it does not exactly consider what Shelford and Clements addressed, namely *coaction*, which is more dynamical than aggregation, and often involves two or more species—even though Allee himself sees the Allee effect as the simplest case of co-operation, hence positive coaction.

**Fig. 2:**
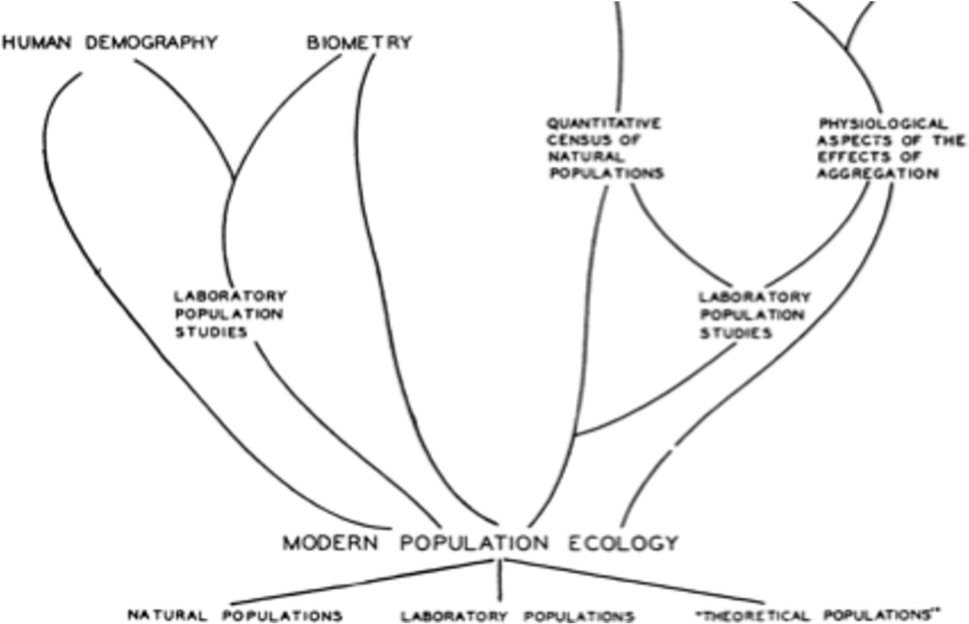
Antecedents of current population ecology according to Park (1946)

In a review paper, Thomas Park later saw aggregation studies as a major theoretical advance for population ecology, since it concerns populations in general.^34^ Of course he cites Allee, who came after “philosophically minded biologists” to tackle the question, as an “ecologist of physiological bent” who did laboratory and field work on it (Park, 1946). This paper, which reflects on Allee’s work, proposes a systematic reflection upon the state of population ecology, which fueled the long section of the *PAE* devoted to the history of the field. Thomas Park proposes a genealogy of population ecology that stems from various branches (Fig. 2). He insists on the fact that relevant differences for ecologists are not between fish populations and bird populations, but between “laboratory”, “natural” and “theoretical” populations (as people in population genetics handle them). Here, the synthesis of various branches of ecology, regarding many studied clades, coalesce in a science likely to deal with populations in general. The *population* is seen as a “level or organization ‘above’ the organism”, and whose understanding is necessary to tackle *communities*. Park indeed sees the “understanding of community structure and function from the viewpoint of its metabolism and energy relationships” (citing the seminal 1942 paper by Lindemann) as the “most important ultimate objective of ecology”. And “studying population” contributes to “this goal”, which is a “major reason” in favor of doing population ecology. Such an epistemological claim thereby inspired the structure of the *PAE*, articulated by the triad organisms/populations/communities. Thus, the *PAE* he wrote with his brother, Clyde Allee, and then Emerson and Schmidt offered a monist ontology and epistemology of such a triadic construction—monist in the sense that these three items appear as instances of the same kind of thing, as I will explain. At the same time, it brought to clarity the distinction, both ontological and epistemological, between *populations* – the object of theoretical studies such as Lotka and Volterra modeling, or researches on aggregation—and *communities*—which define another level and scale of theoretical ecology.

#### Emerson

Noteworthily, Alfred Emerson was among the acknowledged readers of Park’s paper. The importance of evolution and the focus on community dynamics was directly relevant to Emerson’s previous work on insect communities. Initially an entomologist, Emerson studied termite colonies (work summarized in Emerson (1938)). He focused on termite nests, and saw them as a proper object to study evolution since they manifest inherited (hence evolvable) patterns of behavior, and may indicate phylogenetic relations.^35^ In several papers published in the 1930s he built up a theory about insect communities that drew upon previous insights by Wheeler (1911), as well as some of Allee’s work and his own strong interest in evolutionary theorizing.^36^ Emerson was indeed strongly inclined towards evolutionary theory, especially in his appeal to phylogeny to understand termite behavior and nesting (Emerson, 1938). By considering his two 1939 papers and his presidential address to the American Ecological Society in 1941, I will indicate here the basic elements of this view.^37^

Emerson thinks there are evidences in favor of considering animal societies as “superorganisms”. Previously, Wheeler (1911) had extensively highlighted parallel features between ant and bee societies, and organisms (1939a, p. 183), understood in the framework of “emergent evolution” represented by philosophers Broad or Loyd Morgan (Gibson et al., 2013), and which also attracted Clements and Shelford (see above). The existence of casts among termites and bees as well as the division of labor existing between reproducers and workers or warriors justified seeing those colonies as organisms, considering the fact that physiological labor is divided among organisms, and that a high degree of differentiation exists between organs developed by distinct insects (1939a, p. 184). Notice that such division of labor differs highly from Allee’s idea of crowding since the latter assumes similarity between organisms, while the former is about their ecological differences.

Moreover, Emerson pinpointed a clear parallel regarding development of those societies and organisms (1939a, p. 195). This led him to his key concept of “homeostasis” (the direct focus of Emerson, 1956), which comes from physiology, and concerns the progressively established control of external environment by the organism or the colonies, entailing a stabilizing effect (1939b, p. 295).

But Emerson acknowledged many levels of ‘organismality’. In his presidential address to the Ecological Society of America in 1941, he writes that “the concept of organism is complex and levels of biological organization occur. Viruses, cells, multicellular organisms, metameric organisms, metamorphic organisms, colonial organisms, species populations, aggregated populations, cyclomorphic populations, sex pairs, family units, social units and certain types of ecological communities, each represent a level of individuality with organismic attributes” (1943, p. 97). This list refers to both Emerson himself (his 1939a), and cites CS: clearly, Emerson’s reasoning builds on the general organicism designed by this latter work; yet he proposes another explanation for it. In which sense?

What makes social insect colonies into an integrated group aptly called super-organism is for him natural selection: “The evidence points to the conclusion that the adaptive evolution of dynamic integration of the population components of social insects can only be explained *through the natural selection of the entire population unit*” (1939b, my emphasis). Populations in general show regulations that are indeed understandable as effects of selection—‘cannibalism’, according to Emerson (1939b) is such an effect, which maintains ‘population control’ (p. 292). But this status extends more generally beyond social insect colonies t*o populations in general,* which differs from Wheeler’s view, focused on social insects. “Thus, cooperative population systems are natural biological units and not merely human concepts based upon statistical summations and formulae (…). The student of population biology, therefore, is attempting to analyze and synthesize objectively real units” (1939b, p. 295). The concept of *population*, here, plays a major theoretical role, because Emerson applies it to both a multicellular organism as a population of *cells*,^38^ and an insect colony as a population of *organisms*. Since the former—the multicellular organism—is under natural selection, the insect society is the “real unit”, exactly as the human being, as a set of cells, is itself the unit of selection (1943, p. 116). Thus Emerson’s argument acknowledges selection on populations, because selection on individual organisms wouldn’t be enough to account even for some of the individual’s adaptations such as mammary glands: the relevant unit to understand selection here is the family (mother and child) (Emerson, 1943, p. 116, my emphasis).

A population of termites will therefore “display many group adaptations”, that will be effects of selection, such as soldier termites. Hence, insect societies are “giving hints” of what a biology of populations should be. Emerson’s reasoning uses the reference to the relation between vertebrates and protozoans used by CS, but in a different way. The analogy here is between insect colonies (a1) and populations (b1) on the one hand, and vertebrates (a2) and protozoa (b2) on the other hand: (b) is a simpler version of (a), and therefore, any integration in (a) can be inferred in (b). Our inference basis here is the knowledge of the relation between protozoa and vertebrates, which concerns the emergence of multicellularity; what we infer, is the status of a simple population, as displaying some kind of integration. But what is implicit here is that the relation between poles 1 and 2, namely, the fact that the insect society is a structured population, so that its “integration” gives “hints to the investigator of simpler population systems, much as the vertebrate physiologist may suggest interactions occurring in the protozoan.” While CS insisted that communities are to organisms what organisms are to protozoan, Emerson introduces another level, namely the “simple population” (1939b, p. 287). In virtue of the fact that colonies are ‘real units’ of selection, one can understand the regulation of populations in an evolutionary way, and then see some populations such as hymenopteran insects as superorganisms; the next step would be thinking of multispecies *communities* as superorganisms. Here identity of the causes of integration supports this wide applicability of the concept of organism:

The same forces which bring about the integration of the organismic units *within the species* [population] can also be shown to be active *in the ecological community* which, of course, came into existence not long after the origin of life itself. The organism, in a sense, projects itself into the community because so many species in the community are adjusted to special organismic structures and integrative mechanisms. (1939a, p. 200)

The notion of population, a key evolutionary concept, thereby allows Emerson to go from the idea of insect societies as superorganisms, to the idea of a whole community as similar to organisms. A termite colony is a population of termites, and those “cooperative population systems” are biologically “real units” (1939b, p. 295). If this is possible, then a whole community, with individuals of various species, may also be the target of selection along as they are “cooperative systems”. Hence, beyond insect societies, Emerson finally considers “ecological communities”, indicating that they have an interspecific (and not intraspecific) integration, which is what we should explain (Emerson, 1943, p. 115) in order to justify an ecological organicism.

Thus, for Emerson interspecific and intraspecific integration are two kinds of integration, defining two “trends of population evolution” started from the beginning of evolution by natural selection, one towards communities, the other towards families or insect societies, which are a complex version of what simple populations generally are.^39^

One should remember here the distinction made by Gleason between two kinds of communities, species-like and organism-like. The analogy (a/b) above-sketched corresponds to this kind of distinction: the simple population, where individuals are alike and interbreed and interact, corresponds to the ‘species’ version of communities; the social insect colony, integrated by strong functional interaction with division of labour (queen/workers), and therefore, with dominance hierarchies, corresponds to the ‘organism’ version of communities.

While Clements and Shelford based organicism on a reflection on the complex cause-effect relation between habitat on the one hand, and plants and animals on the other hand, mediated by a system of coactions, Emerson introduced organicism via the reference to population, and the complex analogy described above between insect colonies and populations. The key role of the population concept then explains why Emerson sketches his research program in these words: “population biology merges with community ecology”. This claim perfectly mirrors the structure of the forthcoming *Principles* (see below § 3.2).

In contrast CS defined ecology in these words: “Ecology is in large measure the science of community populations.” (CS, 3) Their sense of ‘communities’ was different, it referred to the integration of plants and animals as responding to a shared habitat, while here, communities are understood by Emerson in a complex hierarchy of meanings that involves a reference to insect colonies and to populations as a basic layer. This conceptual architecture will persist throughout the *PAE*.

For Emerson, a crucial consideration at the basis of the shift from populations to communities, and hence from the intraspecific to the interspecific level, is the role of *symbioses* (1939b, p. 296). He speaks in this sense of “mutual *adaptive* interspecific cooperation” (p. 297, my emphasis). With such a reference to “adaptation”, Emerson’s theory thereby connects the integration of populations and communities to the action of selection. This differs from Allee’s account, according to which cooperation and selection were supposed to be distinct and even opposed forces.

For Emerson, the emphasis on evolution is pervasive and clear, and he sees it as a “fifth dimension of ecological study” (1943, p. 99), after the three-dimensional space and the time-dimension of “ecological succession”, which “may be likened to the ontogenetic sequence of individual organisms.” For this reason, Wright is extensively cited by Emerson; especially, his view of selection on integrated genotypes yields the idea that an integrated group can be acted upon by selection (1939a, p. 197). Emerson mentions the parallel made by Wright between the ‘living organism’ and a species, since both ‘depend on balance’, which is always fostered by natural selection (ibid.).

To this extent, he follows Wright in emphasizing that “selection acts upon the organismic unit and not merely upon the germ plasm” (ib., p. 196). Wright (1948) sketched this parallel between the organism and the species, based on ‘protoplasmic continuity’, so that he sees the latter as a “less integrated organism”.^40^ Earlier, Allee had borrowed from Wright a similar conclusion about the role of selection on integrated alleles.^41^ Notwithstanding their differences (see below), Emerson and Allee share the same Wrightian understanding of natural selection as tied with integration, namely, as simultaneously *promoting* integration (through fostering functional integration), and *targeting* integration, i.e., the integrated effects of genes. Hence Emerson sees selection as concerned not by individual effects, but by collective effects of genes.^42^

Noticeably, Emerson’s, (1939a, b) paper is grateful to Clyde Allee and Sewall Wright. In his SBT, Wright crucially considered that selection acts on *demes* (interdeme-selection is the third stage of the SBT process), hence loosely structured groups of same species individuals. “The course of evolution of the species as a whole is then determined by interdemic selection”, he wrote later (Wright, 1960, p. 618). To this extent, Wright’s concept of selection (rather than Fisher’s or some other view) allowed for the idea of a selection acting on ensembles; through Emerson, it became a major brick of the conceptual architecture of the *Principles of Animal Ecology*, and one can safely argue that constant interactions with Wright in Chicago, as well as reciprocal reading of one another’s writings between Wright, Emerson and Allee, partly yielded such architecture.

Population and communities as integrated, rely on natural selection; they can appear as superorganisms through the above mentioned analogy with social insects colonies – rather than through the complex and hierarchical integration of a set of responses to a common habitat, as in CS. On this basis, Emerson can draw important consequences that lean toward organicism. “The fact is fairly clear that ontogenetic development, growth, and death, as well as sexual and asexual reproduction, are remarkably parallel in the organism and the superorganism” (1939a, p. 195). Because of the general applicability of the organism concept, communities and organism do have *development*, as Clements had emphasized; yet the reason for this general applicability is different now: it is *evolutionary*. Like species, communities even undergo evolution: “the ecological community also has an ontogeny (succession) and a phylogeny” (1943, p. 200).

As compared to Allee however, and even though he consistently cites him—especially regarding aggregation of termites (1943, p. 111)—Emerson doesn’t start with crowding, namely a static view of numbers. Instead, he begins by looking at groups of organisms as having different functions, namely different activities, which impinges onto other groups’ conditions of existence via coactions; only then he questions the explanations for integration. But the double analogy indicated above (a1,2/b1,2), centered on insect colonies, indicates that two kinds of collectives can be organism-like: either simple populations, or complex colonies and then communities. Allee’s case, namely crowding and aggregation, can only be subsumed under the former kind of collective.

In the end of the decade, both authors joined forces with the Parks and Schmidt in order to systematize an ecological thinking on those bases; this thinking proposes a detailed and systematic parallel between populations, communities and organisms.

### The principles: a table of correspondances

Communities feature a “homeostasis”, as Emerson named this principle of integration, and this is what allows them to be integrated by coaction rather than disintegrated by disoperation. Homeostasis is a physiological term which applies at various levels of individuality, from organisms to ecosystems through populations. Symbiosis plays an important role in this homeostasis (as emphasized by Emerson), similarly to the pure effects of aggregation (as emphasized by Allee).

The structure of the PAE starts from “Organism”, then addresses “Environment” then “Population”, then “Communities”, then “Evolution and Ecology”. As in Emerson’s thought, the level of ‘*population*’ has a key role in showing that some supra-individual level can be seen as acted upon by selection. Referring to *Bioecology* and its organicism, authors write that “there is some meaning in thinking of the *basic ecology of populations* in the same terms that CS used for *communities*” (PAE, 348).

**Fig. 3:**
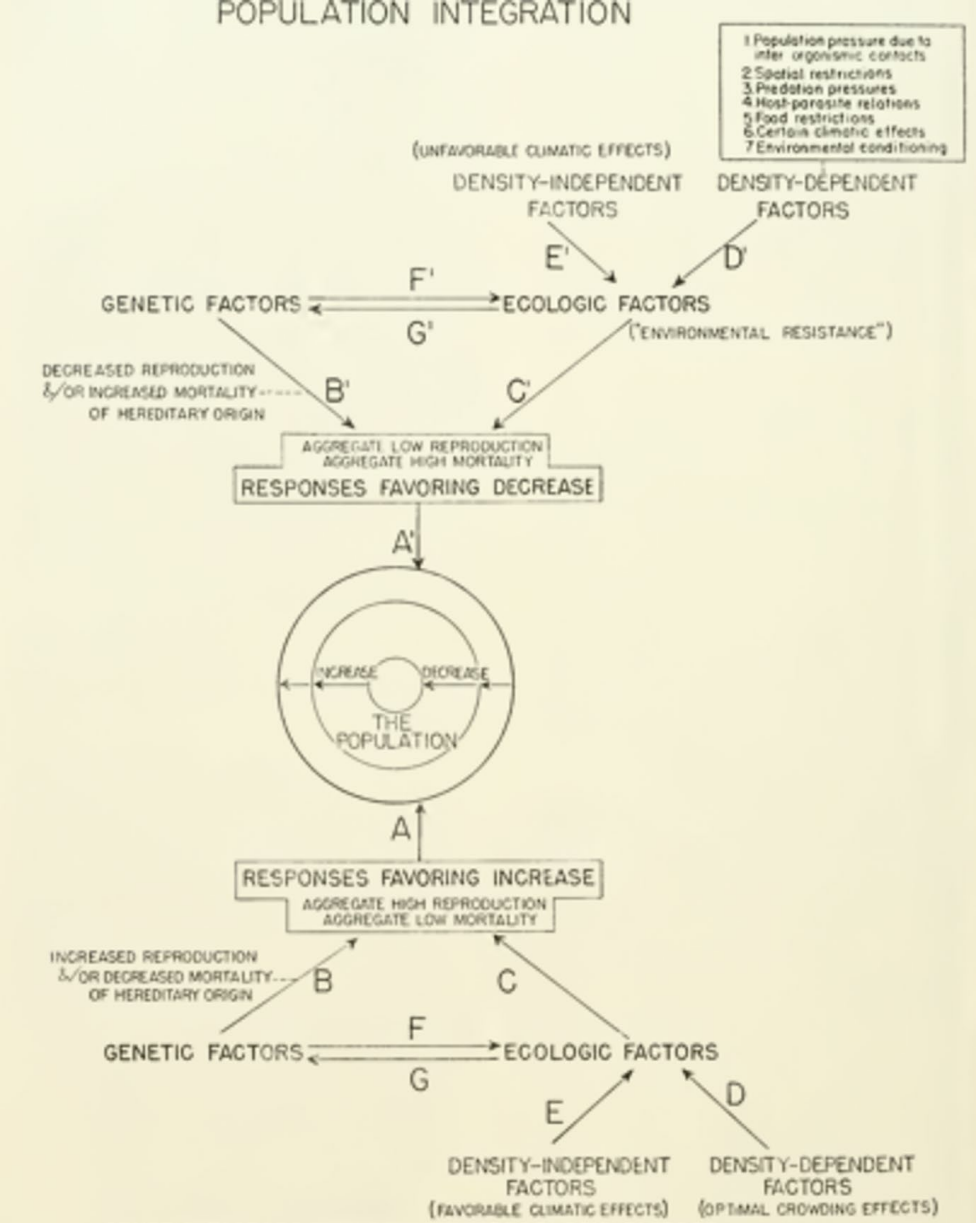
Pressures that yield population integration. (Allee et al., 1949)

Populations are integrated by a set of interactions or ‘pressures’ whose balance produces the positive coaction sought by Clements and Shelford in communities. Allee and colleagues write: “those pressures are statistical because they arise from group phenomena. A particular pressure grows out a particular operation. It merges with another that is closely related. (…) These pressures are integrated in the sense that, as in an organism, change in one affects another and results in some compensatory regulation in the system. There is nothing mystical inherently in this statement. If more data were available about a certain population, it should be possible to express the integration in arithmetical terms” (PAE, 392). Thus the *Principles* see a difference in degree of mathematical tractability and not in nature between populations and organisms with respect to their integration. The Fig. 3 below shows how those pressures can be understood and integrated: they are decomposed into positive and negative pressures (regarding population size), each of them being divided into genetic and ecological factors. The latter are in turn divided between density-dependent and density-independent factors. This last distinction refers to a major controversy among population ecologists, regarding the main reason for population regulation. Some favored processes whose intensity doesn’t depend upon the density of the population, such as climate (Andrewartha & Birch, 1954), others ascribed regulation to processes whose intensity varies with the density of the population, such as competition (especially, Nicholson & Bailey, 1935). This controversy animated ecologists in the following decade, and found a conclusion or conciliation in late 1950s (see Huneman, 2019; Stearns, 1982).

As a result, a population features “five general attributes” shared with organisms: “a definite structure and composition” which “fluctuates with age; “ontogenesis”, hence “growth, differentiation and division of labor, senescence and death”; heredity; integration by “genetic and ecologic factors”; and being a “unit that meets the impact of its environment” and alters it (PAE, 264).

Thus, a population is a unit of selection, just as what Emerson thought. Communities are just a unit of a higher degree, in which integration requires symbioses because of interspecific relations. Communities, like organisms, stand in flux—“organisms die and are replaced by their descendants or ecologically equivalent organisms” (PAE, 439)—but “during this continual activity the community remains relatively stable, and its characteristic aspect and taxonomic composition are substantially unchanged” (ibid). A distinctively Clementsian organicism is visible when the authors add that “communities tend to evolve, under normal conditions, to a highly stable end point, the *climax community*” (ibid, my emphasis).

Thus, “cells, organisms, populations, societies and communities” are “progressively complex biological systems”, which means that they are of the same nature: “all five are protoplasmic interdependent integrations” (PAE, 440). The protoplasmic continuity was, for Wright (§3.1.b above), a reason to think of selection on species, hence PAE borrowed the argument from him, but the object slightly shifted: protoplasmic integration requires organisms and then, because of their pervasive coactions, such integration is prolongated towards communities.

**Fig. 4:**
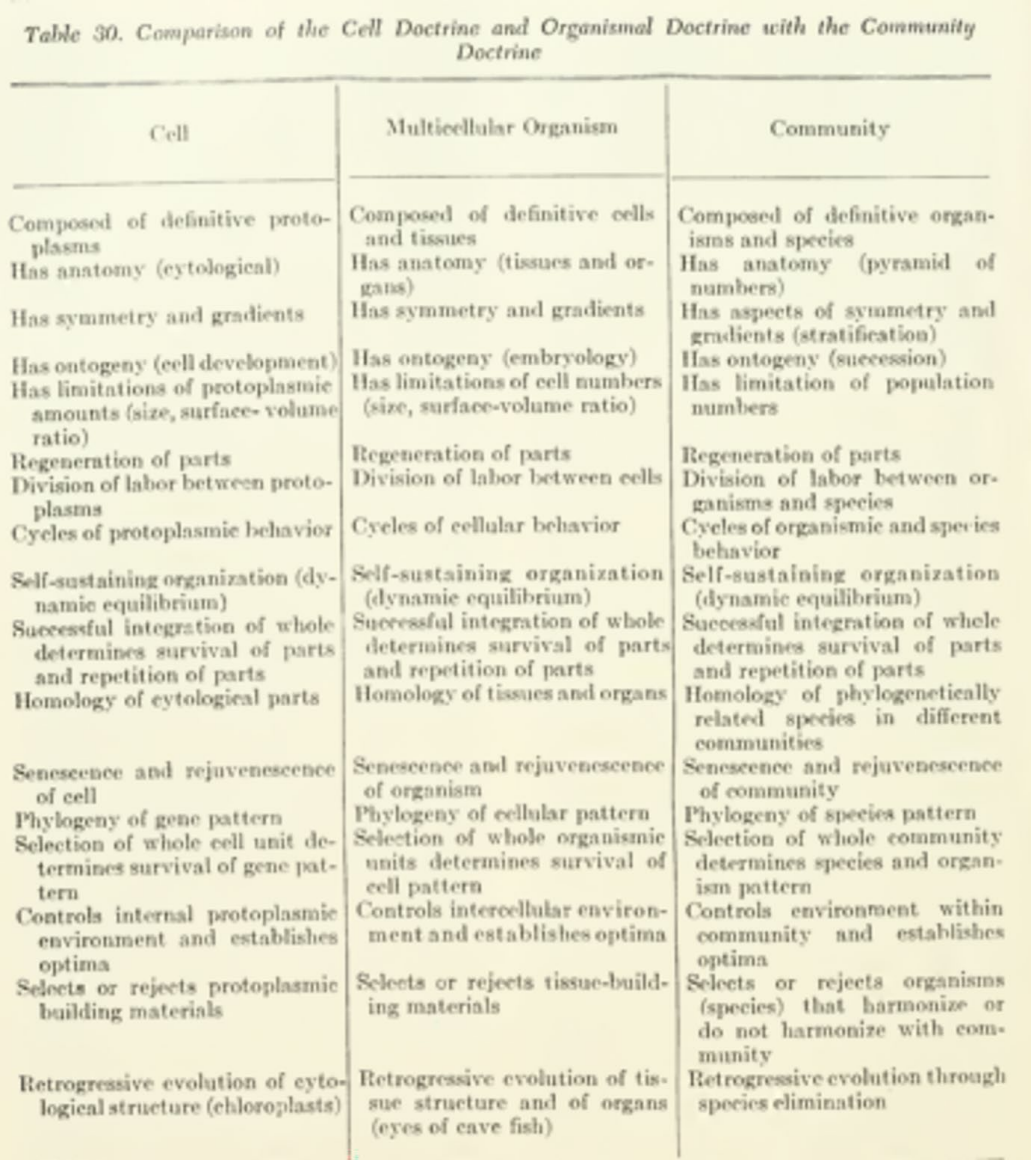
The correspondence table between cells, organisms, and communities. (PAE 440)

Thus, “organisms would tend to form natural groups of foods and feeders—in other words would form communities.” (PAE 437) This process means that communities are not—*contra* Gleason’s individualistic concept—samples of species. PAE explicitly rejects Gleason’s idea of an often stochastic arrangement of species due to the vagaries of changing environments: “communities are composed, not of random assortment of species, but of ecologically compatible species populations whose collective ecological requirements or food, shelter and reproduction are satisfied, in the last analysis, by a certain range of environments” (ibid). Because of the common basis of all integration within protoplasm continuity, this view is called an “extension of the cell doctrine”, and justifies the table of correspondences (p. 440; below Fig. 4). The key ontological claim is that at each level a selection on a whole unit (cell, organism, population, community) determines the survival of a pattern of entities of the lower level.

The doctrine of populations and communities as units of selection, inspired by Wright, and elaborated mostly by Emerson, is formalized here into this expression of the structure of selection. The fact that each selection process acts upon a pattern of entities at a lower level ensures that all selective processes interact together. Then, the generality of this form of the selection process allows one to infer statements and claims across levels. Finally, the notion of ‘pattern’ allows one to link selection to *metabolism*—understood as the maintenance of a specific pattern—and therefore articulates Wright’s idea of selection with Shelfords’ (1931)’s notion of metabolism in communities, defining the specific organicism that the book defends: “The sum total of the organismal nutritional and assimilative responses of the community may be considered to be the metabolism of the community, just as the sum total of the physicochemical processes in the organicism thought of as the metabolism of the individual. In both instances, these metabolic wholes are composed of spatially integrated and stratified processes” (PAE, 495).

The resulting theoretical structure of ecology is the following. “The organism is an essential connecting link”, articulating in the table what is below (cells) to what stands above it (communities): “it is the hinge on which both cells and communities depend for continued existence. In the same way, organismal survival is dependent on cells for assimilation, and on communities for food supply” (PAE, 441).

In turn, the community integration is clearly due to selection, which acts upon communities and populations exactly as upon organisms. First, it targets populations of one species: “Selection operates on parts and wholes of genetically connected intraspecies populations (i.e. species, cyclomorphic populations, sex pairs, family units, and societies) in a manner similar to the action of selection on protoplasmatically connected organisms” (PAE, 694, my emphasis) (and they cite Weissmann). Then, the role of protoplasm allows the authors to assimilate selection on organisms and on populations, directly echoing Wright’s reasoning about species selection summarized above. About integration in communities, PAE cite *Bioecology*: “coaction constitutes the chief bond in the community” (PAE, 698). Along the lines of CS, they add that the “coactive species pair” is the elementary unit of this coaction.

Coactions are therefore the material of natural selection, whose effect may extend beyond species pairs and drive community interaction. “The whole community tends through the process of natural selection operating on complex coactions, to attain a reactive equilibrium sufficient to carry the quantitative pattern of interspecies relations over long periods of time” (PAE, 705). This has many major consequences, for instance, “the food niche becomes an ecological extension of the heredity of the population” (PAE, 707).^43^ While CS saw integration as based on coaction and cooperation, and while Allee previously opposed selection to cooperation, the PAE shifts the focus by *unifying coaction and selection.*

More generally, evolution strengthens mutualist interactions, which integrate species together in a community. For instance, plants coevolve with herbivores: PAE hypothesizes that “plants have slowly become adjusted to the evolution of herbivores through various regenerations and protective devices” (PAE, 705). Yet *antagonistic* interactions too (like predation) evolve towards a coaction that fosters integration of the community.^44^ Finally, the *Principles* generalized across communities the relations between selection, antagonism, cooperation and integration in the following way: “reciprocal evolution of exploited and exploiting forms has occurred, so that the selection pressures through exploitation gradually sort organisms in relation to each other, and that *these evolutionary relationships create highly important interacting, interdependent systems of species*” (PAE, 704 my emphasis).

Those are the main avenues along which natural selection yields community integration, and supports a community-level metabolism described here: “The sum total of the organismal nutritional and assimilative responses of the community may be considered to be the metabolism of the community, just as the sum total of the physicochemical processes in the organicism thought of as the metabolism of the individual. In both instances, these metabolic wholes are composed of spatially integrated and stratified processes” (PAE, 495). This metabolism concept develops within PAE an indication by Park and by Shelford (1931), who saw “food interrelations of animals” as “interaction, which we may call processes comparable to metabolism.”^45^

Such a view allows PAE to explain a ‘homeostasis’ that exists not only in organisms, but also, following Emerson’s use, in populations and communities. The cost of this very general approach is that it can’t provide mathematical modeling of the various factors, unlike Nicholson’s model of density dependent interactions that regulate population numbers, discussed in the last section (PAE, 710). Here one can see all factors that can affect population integration qualitatively in a table, but neither their intensity nor their relations are mathematized.

To sum up, the *Principles* elaborated in a same movement an *ontology* of ecology based on the homology between organisms, populations and communities—and a *methodological* claim about the uniformity of approaches, based on an epistemological principle, namely the search for selectionist explanations of system integration. Thus, the book presented a systematic view of community and population ecology that synthesized a Clementsian understanding of communities as organism-like—informed by studies of social insects such as Wheeler’s—and Wright’s understanding of natural selection as targeting integrated sets of elements and tightening integration.

Organicism is a unifying scheme in twentieth century ecology. For Clements and Shelford, it unified animal and plant ecology, at a time where their disciplinary and philosophical separation obfuscated their methodological unity, and emphasized the holistic essence of communities as plant-animal integrated responses to habitats. As for Allee, Emerson and the others, organicism provides a unifying principle for all levels of analysis, from cells to communities. It is therefore less a claim about ontology than an organizing theoretical principle, as well as a way to define in a level-independent manner some methodological research questions (disoperation and operation, facilitation, coaction…).

### Evolutionary organicism fading away

Allee et al.’s *Principles* are often counted among the many evolutionary studies committed to an idea of group selection that one reads in authors such as Weissmann or Eugene Warming, and whose idea has been renewed later by Wynne-Edwardes (1962) (Gibson et al. 2013, Borrello, 2003), for whom selection aims at the good of the species. Therefore, such groundwork is part of a long term history of group selection understood in the largest sense. But notwithstanding the connection with this long-term history of group selectionism the *Principles* relied on a recent and particular conception of natural selection defended by Wright, and accepted by many, such as Dobzhansky or Mayr—a view that was central in the Modern Synthesis. Nicolson (2016) insisted that ecologists in early 1950s were concerned by being integrated in the Modern Synthesis seen then as a major scientific and institutional achievement. By investing a notion of selection accepted and checked by Wright to reframe Clements’s and Shelford’s organicism, Allee and his colleagues advanced this general integrative aim; though they did it through a pathway soon to be abandoned.

For a current reader, the group-selectionist story put forth by Allee and his colleagues is blatantly wrong. In 1982, writing their biographical memoir about Emerson, E.O. Wilson and Charles Michener characterized his adoption of the superorganism concept—so influential in the *Principes*—in this way: “his method of analogy, first put in concrete form by Wheeler and highly popular in the first half of the century, was perhaps carried to its extreme by Emerson. He saw in the social insects the exemplification of “dynamic homeostasis,” which he believed to be a new unifying principle of evolutionary theory. This part of Emerson's thought has had relatively little impact, principally because during the period of his most assertive articles (1952–1958) the pendulum had begun to swing away from holistic conceptualization and toward piecemeal, experimental analysis of individual physiological mechanisms and patterns of behavior.” (Wilson & Michener, 1982, p. 164, my emphasis). The rest of the story of evolutionary organicism is about this “swinging away.”

The aforementioned debate over density-dependent and density-independent processes as candidate accounts for population regulation raged in the 50s (Kingsland, 1995). To some extent, the *Principles*’ organicism didn’t favor any one of the approaches, precisely because they stood at a very general standpoint. The fact that organicism was trans-scale—including equally population and communities—implied that a same answer had to be given to the two major ecological questions: the one involved in the hot debate—namely, the “natural control” question, or the integration of *populations—*and the species coexistence question, or integration of *communities*. This answer was a notion of “natural selection” tailored to either populations or communities.

But David Lack had proposed in 1947 a density-dependent solution to the “natural control” issue. He has shown that selection was necessarily acting at the level of individuals, not groups. The trait he considered was clutch size, which in birds conditions the reproductive rate just mentioned. Since it directly correlates with the number of offspring, while fitness (which measures evolutionary success) is somehow correlated to such number of offspring, clutch size is a very interesting trait for anyone who wants to study natural selection (Lack, 1947, p. 95).

Yet the *Principles* also considered clutch-size. The difference between the two approaches is significant here. Allee et al. wrote that “in species under natural conditions, there would seem to be an intrinsic psycho-physiological mechanism that maintains a number of eggs characteristic for each species, this number being presumably *optimal for the species* under the given condition” (PAE, 701, my emphasis). Lack, in his 1947 paper, also used the notion of optimum, writing: “for broods above the average size, proportionate mortality among the young would rise as brood size increases [.. then] a point is reached when an increase in the number of eggs is offset by the increase in mortality, so that there is no increase in the number of young raised.” This amount is determined by selection; then Lack claims: “the commonest brood-size found in nature is also the size *with optimum productivity*” (*ibid*, my emphasis). But, while PAE saw the optimum as determined by species selection, Lack shifted the meaning of selection.^46^

So he explicitly denies that the reproductive rate, hence clutch-size, is “adjusted by natural selection to balance the mortality of the species” (1954, p. 8), whereas this is exactly Allee et al.’s final claim. Such idea, he says “rests on a mistaken view of both population balance and natural selection.” Lack’s reasoning is the following: because natural selection “operates on the survival rate of each individual or genotype”—fitness being so defined -, then, if a larger clutch-size were to appear it would invade the population except if “for some unknown reasons the individuals laying more eggs leave fewer, not more, descendants” (Lack, 1954, p. 22). This unknown reason is precisely what his 1947 enquiries about birds established, namely that after some point—where “an increase in the number of eggs is offset by the increase in mortality” (1947)—one more nestling decreases the survival rate of all offspring. Everything occurs at the level of the individual and its offspring. Selection above the level of the individual here plays no role to understand the clutch-size, *contra* the *PAE's* account.

The rise of density-dependent approaches to population regulation provided another way to understand how selection regulates numbers, and this does not include any group selection of the form needed to ground the organicism presented in the Correspondence Table of the *Principles*. The adoption of Lack’s views, and, later on, of what came to be known as behavioral ecology, made the *Principles*’ organicism irrelevant as a framework to do animal ecology. In the *Principles,* selection sensu Wright (interdemic, targeting integrated collectives) justified organicism about populations and communities. If selection is proved to target individuals and never collectives, then the justification for organicism fades away. This opens a way for an account of associations that is not community-based, not holistic—in other words, Gleason’s individualistic concept of associations. The *Principles* shifted Clementsian organicism from physiology towards evolution, through using Wright’s concept of (interdemic) selection. Once natural selection changes meaning by concentrating on individuals, this is no more possible (see also Grodwohl 2019 on behavioural ecology in the Modern Synthesis).

## Conclusion

Organicism in ecology is often seen as the idea that ecosystems or communities are organisms. For many, it has roots in German *Naturphilosophie* (e.g. Tobey, 1981), or more generally in a romantic view of microcosm analogies between individuals and the universe. The conceptual grid to address an ecosystem therefore comes from physiology, as the science of the organism, even though one can distinguish one more holistic and one more analytic organicism (mutualism vs. dominance as Kirchhoff (2020) reconstituted it). This accounts for the anti-scientific aura that nowadays accompanies organicism; the reception of the Gaia hypothesis testifies how this reputation is pregnant (Dutreuil, 2024).

Here, I examined a kind of organicism that pervaded the first half of the twentieth century in ecology, and that does not pertain to any kind of romanticism. Eliot (2005) after Hagen (1988) indeed did justice to the reputation of romanticism assigned to Clement’s holism, reminding us that he was before all an adept of causal investigations in an experimental analytic manner. Organicism was a major part of two of the scientific syntheses proposed, around 1940 in 1950 in theoretical ecology—namely *Bioecology* and the *Principles*. I have shown that while the former designed an organicism based on physiological and developmental concepts, Allee and his colleagues shifted from physiology to evolution by natural selection. I thought of their conceptual scheme as the Wright-Clements connection; the roots of this ecologists’ team at Chicago and their personal connections has been decisive here. At the dawn of the fifties, Darwinian evolution—mostly stemming from Wright—grounded a major organicist synthesis in ecology, in which not only communities, but also populations, are superorganisms. This advent has been prepared for decades, especially through Wheeler’s seminal paper on superorganisms and insects, Allee’s studies on aggregation, Emerson’s studies on termites, and therefore a general metaphysical background in line with philosophers of ‘emergent evolution’ in the 1920s. I finally argued that the clash between this view and the understanding of density-dependent regulation processes led to the demise of the Wright-Clements connection, and the end of the first Darwinian organicism in ecology.

## Footnotes

1 For the complex story of the various introductions of Modern Synthesis evolutionary theory in ecology, see Huneman (2019).

2 On réduction and emergence see Sartenaer (2016); on individualism and holism, see for instance Epstein (2018). On mechanicism and organicism, see among others Ginsborg (2004) Huneman (2006, forth.).

3 The notion of function is complex, and not the topic here; for the various meanings see Gayon et al. (2023).

4 See Nicolson (1989); I thank Antoine Dussault for this information.

5 See also Kirchhoff (2020).

6 “Since Elton’s treatment of communities is without questions the best of the decade, we can do no better than examine the state of this phase of ecology as seen through his eyes” (PAE, 58). See Dussault (2020), on Elton’s animal ecology.

7 The authors write: “Plant ecology confines itself primarily and exhaustively with the cause-and-effect relation between the habitat on the one hand, and the organism and the community on the other. All further relations arise out of this, and all other approaches are incomplete unless they lead back to it. With the inclusion of animals in the biotic formation (biome), this relation naturally becomes more complex, but it is nonetheless valid.” (CS, 3) This is in accordance with Clements’ constant concern with unraveling causal relations in ecological settings, as established by Eliot (2005).

8 I thank an anonymous reviewer for this remark.

9 “[Developmental relations] were stated to be very generally in agreement. [Any] Disagreement was said to be temporary, and to accompany rapid successional changes. Succession was stated to be due to an increment of changes in conditions produced by the plants and animals living at a given point” (CS, 8).

10 “In their study of communities in the San Juan Channel of Puget Sound, Shelford and Towler found general the principles drawn from plant communities by Clements to be readily applicable. The analysis yielded three benthic formations or climaxes with two associations each and a number of more or less definite seral stages” (CS, 18).

11 They cite his *Physiological animal geography* (1911), which says: “There are two distinct points of view for biological investigation. One is that of evolution; the other, that of physiology, or the explanation of the organism in terms of physics and chemistry. One may make a physiological explanation of the behavior or structure of an organism and in no way explain its evolution. On the other hand one may make an evolutionary explanation of an organism without making any contribution to its physiology. (…) We are concerned with the physiological relations of animals to natural environments.”

12 See Ilerbaig (1999) on Shelford’s move from evolution to physiology.

13 Aristotelians called this ‘proportionality analogy’, see references in Huneman (2007a, 2007b).

14 On these emergentists see Sartenaer (2015).

15 This is all the more interesting that later someone in the tradition of Hutchinsonian niche ecology—for instance Tilman's limiting similarity view of species distributions in communities—could understand the habitat-community causal relation in terms of selection, since the simplest Darwinian schema (termed ‘externalism’ by Godfrey-Smith, 1996) sees the object to explain as caused by the environment (namely, the habitat) through natural selection. But precisely, CS don’t go this path at all.

16 On this debate, see Kingsland (1995), Huneman (2019). This discussion on the word ‘factor’ directly anticipates the debates over whether to use ‘causes’, ‘forces’ or ‘factors’ to conceive of what drives evolution (i.e. selection, mutation, migration, drift) (see Depew, 2013).

17 “This is notably true of aggregation, in which the consequences may be beneficial or injurious to the species concerned, or the two may be combined in varying degree. When the first outweighs the second, the result is *cooperation* in some measure; if the scales are reversed, the outcome may well be termed *disoperation*. This comprises several types of which competition is the most important, ranging from mere subordination or displacement at one extreme of effect to complete destruction at the other” (CS, 106, my emphasis).

18 “The universal role of coaction is to be seen in the integration of plant and animal relations to constitute an organic complex, which is characterized by a certain degree of dynamic balance in numbers and effects” (CS, 107).

19 I thank a reviewer for asking this question.

20 The notion anticipates what is now called ‘ecosystem engineering’ by current ecologists (see Jones et al., 1994).

21 “It is obvious that the coaction of coming together in a family or other group will set up other coactions as corollaries of this, and each may be of greater or less significance in the life of the community” (CS, 149).

22 “If, as is insisted, the community is an organism, it should be possible to study the metabolism of that organism” (p. 268). I thank Antoine Dussault for this reference.

23 The preface states: “The appearance of Professor Richard Hesse's *Tiergeographie auf oekologischer Grundlage* in 1924 marked the beginning of the new phase in the development both of ecology and of animal geography in the latter field it made the first serious attempt to apply ecological methods, principles, and facts to the study of animal distribution in a world wide scale.”

24 According to Gibson et al. (2013, p. 513)Wheeler was “the nation’s foremost advocate for emergent evolution during the late 1920s.”

25 Coactions “influence the population (or community) through (1) “cooperation”, which has survival value for at least a majority of the cooperating organisms; (2) “disoperation”, which has deleterious effects on the coacting organisms, either through influence on the habitat, on contiguous group members, or both; and (3) “competition, which is an expression of the fact that certain coactions are directed towards exploiting an environment limited in its potentialities. Competition may be either favorable or unfavorable in terms of long- range results” (PAE, 348).

26 See Mitman (1992), and Schmidt (1957) for the contextual interpretation of Allee’s work—as a quaker, finding out principles of mutual aid and benevolence within groups was a substantial continuous motivation for him. He constantly thought of the relevance of his animal studies for human beings, and saw them as showing a natural bent towards sociality.

27 “An initial population of 4 *Tribolium* beetles per 32 g of flour reproduces more rapidly during the 25 days following than smaller or larger initial populations (Park, 1948). (…) The benefits resulting from an increase in the size of aggregations up to the optimum represents cooperation; the harmful effects resulting from aggregations that are too large is disoperation “(ibid)” (Kendeigh, 1961, p. 175).

28 At the same time, notice that Elton would oppose two factors driving ecological dynamics, namely natural selection on the one hand, and an unconscious drive through which animals chose their habitat (Elton, 1930, analyzed in Huneman, 2019).

29 Caron (1977) analyzes Allee’s evolution with respect to the interpretation of crowding as a progressive integration of a modern evolutionary viewpoint, and thus involving a higher role ascribed to selection of interacting groups. My analysis ascribes a more precise role to Wright’s account.

30 In a nutshell the SBT is verbally summarized by Allee in this way: “if a species occurs not as a single breeding unit but broken into effective breeding colonies which are almost isolated from each other, the members of different colonies, given sufficient vigor, may evolve into dissimilar local races. If one of these becomes well adapted to its environment it may increase in numbers and send out numerous emigrants. If these emigrants find and interbreed with members of other less advanced colonies they will grade these up until they resemble the most adapted colony” (Allee, 1938,  p. 127).

31 Therefore “with the species population intermediate in size, with the same mutation and selection rates as before, gene frequencies move about at random but without reaching the degree of fixation found in the [case of large population]. Since it will *be easier to escape from low adaptive peaks*, the population will tend finally to occupy the more adapted levels” (Allee, 1938, p. 130). Allee emphasizes (highlighted sentence) exactly the core of the SBT target problem—how can a population leave local adaptive optima? In characterizing populations of this intermediary size, Allee even uses Wright’s metaphorical geographical language: “under these conditions the chances are good that some of the local colonies will escape from the influence of Mount Minor Adaptation and manage to cross the valley to Mount Major Adaptation. Here the race will expand in numbers and will send out more and more emigrants which will interbreed with the stocks in the less adapted colonies and tend to grade them all up toward a higher adaptive level” (ib., p. 130).

32 Allee uses an unknown or invented quotation of Wright to back it up: “The conclusion is as Professor Wright says: ‘A subdivision of a large species into numerous small, partially isolated races gives the most effective setting for the operation of the trial and error mechanism in the field of evolution that results from gene combinations'” (id. p. 131).

33 For instance the acknowledgements of Allee (1938) note: “the kindly criticism of Professor Alfred E. Emerson has been particularly helpful in developing the work and in shaping the content and implications of these lectures. My thanks are given also to Professor Sewall Wright for his criticism of Chapter IV” (14). Allee (1932) was “critically“ read by Shelford, and Emerson corrected two chapters of Allee (1931).

34 “Aggregation studies had a considerable effect on the growth of population ecology. (…) these studies with their emphasis on crowding led to further consideration of population density from the viewpoint of general ecology but frequently studied experimentally” (Park, 1946).

35 “Termite nests may be used as examples of behavior evolution because they are morphological indications of behavior patterns, they express the behavior of a population, the patterns are hereditary, there is a natural control over any Lamarckian influence, evolutionary sequences are available, adaptive modifications may be demonstrated, and coordination mechanisms may be partially analyzed” (Emerson, 1938, p. 281).

36 On Emerson’s views of communities and their relations with Wheeler see Gibson et al. (2013).

37 See Mitman (1988) on Emerson and group selection, though the focus here is different.

38 Notice that Emerson was part of the discussion of Gleason (1939) paper; there, he remarked that “the individual is a population of cells” (Gleason 1939, p. 110).

39 “Two trends of population evolution emerged in the development of greater living efficiency by the means of natural selection. Ecological communities developed with interspecific integration which produced a partial environmental control and a relative environmental stability for the individual organism. In addition to this type of organization, a second evolutionary trend directed the development of intraspecific population groups and progressed towards greater and greater control of an otherwise fluctuating environment” (Emerson, 1943).

40 “Evolution above the level of the bacteria and the blue green algae is largely concerned with a loosely knit entity, the species. The species may be looked upon as an organism with literal protoplasmic continuity in the space–time of the physicist, through the continual union of lines of germ cells. In many animals this integration is supplemented by that based on social behavior. (…) The individuals are the ever changing front with which the species confronts its environment. But while above the individual from the standpoint of composition, the species is incomparably less integrated organism” (1948, p. 538). The first sentence echoes Wright’s seminal paper on “Evolution in Mendelian populations”: “As to evolution, its entities, species and ecologic systems, are less closely knit than individual organisms” (1931, p. 156).

41 While considering SBT he notes: “random differentiation of local populations furnishes material for the action of selection on types as wholes, rather than on the mere average adaptive effects of individual genes” (Allee 1938, p. 127).

42 “Selection probably does not act upon the single effect, but selects genes and gene combinations which result in a sum total of favorable effects. Consequently a gene or system of genes causing degeneration may be selected for other beneficial effects, particularly if a former beneficial effect has been lost or decreased through a change in the environment” (Emerson, 1938, p. 280).

43 One cannot but notice that the formulation predates precisely the notion of ‘ecological inheritance’ that has been put forth by advocates of niche construction (Odling-Smee et al., 2003) twenty yeas ago.

44 “The food relations within the food web are usually the result of long evolutionary action. For example, insects whose food is limited to a species or higher taxonomic category of plant are usually adjusted to special biochemical traits of their hosts, and therefore have evolved a coactive relationship” (PAE, 699).

45 I am grateful to Antoine Dussault for this reference.

46 Hagen 1992 (chap. 8) or Collins (1986) consider Lack’s view of selection and its contrast with group selectionism, while Borello discusses the way Wynne-Edwardes attempted to debunk this view. He is for this reason credited to have fathered evolutionary ecology, for instance by Anderson (2013) or by Kimler, (1986) or in Orians’s (1962) appraisal of the density-dependance debate. Yet I emphasized the contrast between a deeply evolutionary view, which is the PAE’s approach, rooted in a major version of the Modern Synthesis (Wright, Dobzhansky), and which took the form of an organicism—and another evolutionary view, which is incompatible with any organicism.
